# BMP9‐ID1 signaling promotes EpCAM‐positive cancer stem cell properties in hepatocellular carcinoma

**DOI:** 10.1002/1878-0261.12963

**Published:** 2021-05-02

**Authors:** Han Chen, Kouki Nio, Taro Yamashita, Hikari Okada, Ru Li, Tsuyoshi Suda, Yingyi Li, Phuong Thi Bich Doan, Akihiro Seki, Hidetoshi Nakagawa, Tadashi Toyama, Takeshi Terashima, Noriho Iida, Tetsuro Shimakami, Hajime Takatori, Kazunori Kawaguchi, Yoshio Sakai, Tatsuya Yamashita, Eishiro Mizukoshi, Masao Honda, Shuichi Kaneko

**Affiliations:** ^1^ Department of Gastroenterology Kanazawa University Hospital Japan; ^2^ Department of General Medicine Kanazawa University Hospital Japan; ^3^ Innovative Clinical Research Center Kanazawa University Japan

**Keywords:** BMP receptor inhibitor, BMP9‐ID1 signaling, cancer stem cells, EpCAM, hepatocellular carcinoma

## Abstract

The malignant nature of hepatocellular carcinoma (HCC) is closely related to the presence of cancer stem cells (CSCs). Bone morphologic protein 9 (BMP9), a member of the transforming growth factor‐beta (TGF‐β) superfamily, was recently reported to be involved in liver diseases including cancer. We aimed to elucidate the role of BMP9 signaling in HCC‐CSC properties and to assess the therapeutic effect of BMP receptor inhibitors in HCC. We have identified that high BMP9 expression in tumor tissues or serum from patients with HCC leads to poorer outcome. BMP9 promoted CSC properties in epithelial cell adhesion molecule (EpCAM)‐positive HCC subtype via enhancing inhibitor of DNA‐binding protein 1 (ID1) expression *in vitro*. Additionally, ID1 knockdown significantly repressed BMP9‐promoted HCC‐CSC properties by suppressing Wnt/β‐catenin signaling. Interestingly, cells treated with BMP receptor inhibitors K02288 and LDN‐212854 blocked HCC‐CSC activation by inhibiting BMP9‐ID1 signaling, in contrast to cells treated with the TGF‐β receptor inhibitor galunisertib. Treatment with LDN‐212854 suppressed HCC tumor growth by repressing ID1 and EpCAM *in vivo*. Our study demonstrates the pivotal role of BMP9‐ID1 signaling in promoting HCC‐CSC properties and the therapeutic potential of BMP receptor inhibitors in treating EpCAM‐positive HCC. Therefore, targeting BMP9‐ID1 signaling could offer novel therapeutic options for patients with malignant HCC.

## Graphical abstract

BMP9‐ID1 signaling plays a pivotal role in promoting the cancer stem cell properties of EpCAM+ hepatocellular carcinoma (HCC) cells by activating Wnt/β‐catenin signaling. Treatment with BMP receptor inhibitors that block BMP9‐ID1 signaling could potentially be considered as targeted therapy for patients with malignant HCC.

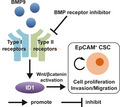

**Abbreviations**
BMP9: bone morphogenetic protein 9
CSC: cancer stem cell
DMEM: Dulbecco's modified Eagle medium
EpCAM: epithelial cell adhesion molecule
HCC: hepatocellular carcinoma
ID1: inhibitor of DNA‐binding protein 1
IHC: immunohistochemistry
LEF: lymphoid enhancer‐binding factor
TCF: T‐cell specific transcription factor
TCGA‐LIHC: the Cancer Genome Atlas Liver Hepatocellular Carcinoma
TGF‐β: transforming growth factor‐beta

## 1 Introduction

Hepatocellular carcinoma (HCC) is a fatal malignant tumor with a high recurrence rate and chemoresistance. The major malignant phenotypes of cancer, including recurrence, metastasis, and chemoresistance, are thought to be attributable to the presence of cancer stem cells (CSCs), and thus, CSCs are now considered to be a pivotal target in cancer therapy [1, 2]. Accordingly, to improve the prognosis of HCC patients, the development of novel therapeutic options targeting the molecular signaling pathways that maintain and/or activate CSCs is desired [3, 4]. We previously classified HCC into two distinct subtypes based on the expression of epithelial cell adhesion molecule (EpCAM) and CD90, which have already been considered as CSC markers in HCC [5]. These HCC subtypes showed distinct tumor phenotypes: EpCAM‐positive (EpCAM+) HCC was characterized by a high tumor growth, invasiveness, and chemoresistance to cytotoxic agents, while CD90‐positive (CD90+) HCC was characterized by high metastatic potential. This suggests that in order to eradicate CSCs, it is necessary to explore and target the associated signaling pathway of each HCC subtype.

Bone morphologic proteins (BMPs) are members of the transforming growth factor‐beta (TGF‐β) superfamily and are the major molecules involved in liver development [6]. Because CSCs share similar features with normal stem cells, including activated biomarkers and signaling pathways, BMP signaling is potentially a pivotal signaling pathway in the regulation of HCC‐CSCs [7]. Among BMP family members, BMP9 has recently been highlighted to be related to stem cell differentiation, angiogenesis, and tumor growth in the liver [8]. Although it has been reported that BMP9 promotes proliferation and exerts an anti‐apoptosis effect in HCC cells [9, 10], the roles of BMP9 signaling in CSC properties still remain unclear.

Here, we demonstrate the potential role of BMP9 and its target transcription factor inhibitor of DNA‐binding protein 1 (ID1) in the activation of EpCAM+ CSC properties in HCC, and we demonstrate the effect of BMP receptor inhibitors, which suppress HCC tumor growth by inhibiting BMP9‐ID1 signaling.

## 2 Materials and methods

### 2.1 Clinical samples

A total of 54 resected HCC tissue specimens (cohort 1) and a total of 37 HCC patient serum samples (cohort 2) were obtained following informed consent from patients who underwent liver resection at the Liver Disease Center of Kanazawa University Hospital from 2010 to 2017. All HCC patient serum samples were collected preoperatively. Cohort 1 and 2 were not completely independent cohorts; 27 patients were included in both cohorts. The study was conformed to the standards set by the Declaration of Helsinki and approved by the institutional review board of the Graduate School of Medical Sciences, Kanazawa University (2017‐323).

### 2.2 Cell lines and reagents

The HCC cell lines Huh7, Huh1, HLE, HLF, and SK‐Hep‐1 were obtained from the Japanese Collection of Research Bioresources Cell Bank (Osaka, Japan) or the American Type Culture Collection (Manassas, VA, USA). The HCC cell line MT was established from resected HCC specimens as described previously [11]. The cells were maintained in Dulbecco's modified Eagle medium (DMEM; Gibco, Grand Island, NY, USA) supplemented with 10% FBS (Gibco) at 37 °C. The BMP receptor inhibitors K02288 and LDN‐212854 and the TGF‐β receptor inhibitor galunisertib were purchased from Selleck Chemicals (Houston, TX, USA) and dissolved in dimethyl sulfoxide. The selectivity of the inhibitors is shown in Table S1.

### 2.3 RNA interference and plasmid transfection

Silencer™ Select ID1‐specific siRNAs s7104 and s7106, and Silencer™ Select negative control siRNA were purchased from Invitrogen (Carlsbad, CA, USA). Cells were cultured in culture medium without FBS for 24 h prior to siRNA transfection. The siRNA constructs were transfected using Lipofectamine RNAiMAX (Invitrogen) in accordance with the manufacturer's protocol. At 4–6 h post‐transfection, cells were washed with PBS to completely remove siRNA constructs in medium and were then cultured in DMEM with 10% FBS.

pCMV6‐AC‐GFP‐ID1 (RG202061) was purchased from OriGene Technologies, Inc. (Rockville, MD, USA), and the pcDNA3.1 (V790‐20) plasmid, which was used as an empty vector control, was purchased from Invitrogen. The DNA constructs were transfected using Lipofectamine 2000 (Invitrogen) in accordance with the manufacturer's protocol. At 4–6 h post‐transfection, cells were washed with PBS to completely remove DNA constructs in medium and were then cultured in DMEM with 10% FBS.

### 2.4 Dual‐luciferase assay

For monitoring the activity of Wnt/β‐catenin signaling pathway in the cultured cells, the pGL4.49 that contains a T‐cell‐specific transcription factor (TCF)‐lymphoid enhancer‐binding factor (LEF) response element and the pRL Renilla Luciferase Control Reporter Vector pRL‐TK was purchased from Promega Co. (Madison, WI, USA). pGL4.49 was transfected using Lipofectamine 2000 in HCC cells growing to 70% confluence along with pRL‐TK following siRNA transfection. One day after vector transfection, cells were treated with BMP9 for 24 h. At 48 h post‐transfection, cell lysates were collected and the luciferase activity was measured using the Dual‐Luciferase Reporter Assay System (Promega) according to the manufacturer's protocol. The luciferase activity of pRL‐TK served as internal control.

For monitoring the promoter/enhancer activity of EpCAM gene, 2151 bp promoter/enhancer fragment of EpCAM gene was inserted into the restriction enzyme site of Xho1/BamH1 of the pGL4.10 (Promega) according to the protocol of the Infusion Cloning Kit (Takara Bio Inc., Kusatsu, Japan). The promoter/enhancer fragment of EpCAM gene was amplified by following primer set: forward: CTCGCTAGCCTCGATCAGATCTCGAGCTCAAGC and reverse: CCGGATTGCCAAGCTGTACCGTCGACTGCAGAA using Prime Star MAX DNA Polymerase (Takara Bio Inc.). pGL4.10 and pRL‐TK were co‐transfected with ID1 siRNA using Lipofectamine 2000 in HCC cells growing to 70% confluence. At 48 h post‐transfection, cell lysates were collected and the luciferase activity was measured using the Dual‐Luciferase Reporter Assay System (Promega) according to the manufacturer's protocol.

### 2.5 Real‐time quantitative PCR

Total RNA was extracted using High Pure RNA Isolation Kit (Roche Diagnostics K.K., Tokyo, Japan) according to the manufacturer's instructions. Quantitative PCR probes EpCAM (Hs00158980_m1), Endoglin (Hs00923996_m1), SMAD1 (Hs00195432_m1), ID1 (Hs03676575_s1), TGF‐β1 (Hs00171257), and SNAI2 (Hs00950344_m1) were purchased from Applied Biosystems (Foster City, CA, USA). The expression of selected genes was determined in triplicate using the 7900 Sequence Detection System (Applied Biosystems). Each sample was normalized relative to the expression of reference genes (β‐actin or 18sRNA).

### 2.6 Serum BMP9 detection

Human BMP9 DuoSet ELISA Kit (DY3209; R&D Systems, Minneapolis, MN, USA) was used for the detection of serum BMP9 in HCC patients in accordance with the manufacturer's instructions. The median value of serum BMP9 (600 pg·mL^−1^) was defined as the objective cutoff value dividing BMP9 high and BMP9 low.

### 2.7 Western blotting

Cell lysates were extracted using radioimmunoprecipitation assay lysis buffer as previously described [12]. The following primary antibodies were used for western blotting: anti‐BMP9 polyclonal, ab 137567 (Abcam, Cambridge, UK); anti‐EpCAM polyclonal, ab71916 (Abcam); anti‐ID1 polyclonal, sc‐133104 (Cell Signaling Technology, Danvers, MA, USA); anti‐β‐actin monoclonal, #4970 (Cell Signaling Technology); anti‐c‐Myc monoclonal, sc‐42 (Santa Cruz Biotechnology, Dallas, TX, USA); anti‐β‐catenin monoclonal, #9562 (Cell Signaling Technology); anti‐non‐phospho(active) β‐catenin monoclonal, #8814 (Cell Signaling Technology); anti‐Phospho‐Smad1/5 monoclonal, #9516s (Cell Signaling Technology); and anti‐Smad5 monoclonal, #12534 (Cell Signaling Technology). Immune complexes were visualized using enhanced chemiluminescence detection reagents (Amersham Biosciences Corp., Piscataway, NJ, USA) according to the manufacturer's instructions.

### 2.8 Immunohistochemistry staining

Formalin‐fixed, paraffin‐embedded tissues were used for immunohistochemistry (IHC) staining. Following deparaffinization, rehydrating, antigen retrieval, and protein block (Protein Block Serum‐Free; Dako, Carpinteria, CA, USA), primary antibodies were applied to slides and incubated at 4 °C overnight. Envision+ Kits (Dako) was used for the qualitative identification of primary antibodies in accordance with the manufacturer's instructions. The following primary antibodies were used for IHC staining: anti‐BMP9 polyclonal, ab35088 (Abcam); anti‐ID1 sc‐133104 (Cell Signaling Technology); anti‐EpCAM polyclonal, ab 71916 (Abcam); and anti‐CD90 monoclonal, ab133350 (Abcam). IHC staining images were obtained using inverted microscopy (Axio Observer Z1; Zeiss, Oberkochen, Germany).

### 2.9 Cell proliferation assay

For cell proliferation assay, single‐cell suspensions of 2.0 × 10^3^ cells were seeded in 96‐well plates, and cell density was evaluated at 24 or 48 h after seeding using the Cell Counting Kit‐8 (Dojindo Laboratories, Kumamoto, Japan) in accordance with the manufacturer's instructions.

### 2.10 Spheroid formation assay

Single‐cell suspensions of 10^3^ cells were seeded in 6‐well Ultra‐Low Attachment Microplates (Corning Costar, New York, NY, USA) after siRNA or plasmid transfection. The diameter of spheroids and the number of spheroids with a size exceeding 200 µm were measured 14 days after seeding. In *ex vivo* limiting dilution assay, 2.0 × 10^2^ or 2.0 × 10^3^ cells obtained from mouse xenografts were seeded in 6‐well Ultra‐Low Attachment Microplates. The diameter of spheroids and the number of spheroids were measured 14 days after seeding.

### 2.11 Transwell invasion/migration assay

Transwell invasion/migration assays were performed using BioCoat Matrigel Invasion Chamber, Cell Culture Inserts, and Control Inserts (Corning) in accordance with the manufacturer's protocols. For invasion/migration assay, 4 × 10^4^ to 10^5^ cells were seeded in the insert chambers and cultured at 37 °C for 24 h. The membrane of the insert chambers was fixed by ice‐cold methanol and stained by hematoxylin and eosin.

### 2.12 Colony formation ability assay

Single‐cell suspensions of 2.0 × 10^3^ cells from mice xenografts were seeded in 6‐cm plates. The colonies were stained by crystal violet at 14 days after seeding, and the number of colonies (> 50 µm) was counted.

### 2.13 Flow cytometry analysis

Harvested cells were resuspended in Hank's Balanced Salt Solution (Lonza, Basel, Switzerland) supplemented with 1% HEPES (Gibco) and 2% FBS. Cells were incubated with FITC‐conjugated anti‐EpCAM antibody (Dako) or FITC‐conjugated anti‐CD90 antibody (Miltenyi Biotec, Bergisch Gladbach, Germany) on ice for 30 min. Labeled cells were analyzed with a flow cytometer FACSCalibur (BD Biosciences, San Jose, CA, USA). Flow cytometry data were analyzed using flowjo™ Software V.10 (BD Biosciences).

### 2.14 Animal studies

NOD.CB17‐*Prkdc^scid^*/J (NOD/SCID) mice were purchased from Charles River Laboratories, Inc. (Wilmington, MA, USA). Mice were housed under specific pathogen‐free conditions with a 12‐h light/dark cycle and provided *ad libitum* access to tap water and food. Huh7 or MT cells (10^6^ cells) were resuspended in 200 µL of a 1 : 1 DMEM : Matrigel (BD Biosciences) mixture and subcutaneously injected into 4‐ to 6‐week‐old NOD/SCID mice. Once tumors had reached a measurable size, mice were randomly divided into two groups (each *n* = 3 or *n* = 5) and intraperitoneally injected with PBS or 6 mg·kg^−1^ LDN‐212854 twice daily for 10–14 days. The size of subcutaneous tumors was recorded every other day. The experimental protocol was approved by the Kanazawa University Animal Care and Use Committee and conformed to the Guide for the Care and Use of Laboratory Animals prepared by the National Academy of Sciences.

### 2.15 Analysis of the Liver Hepatocellular Carcinoma dataset

Using the cBio Cancer Genomics Portal [13], the data of the Cancer Genome Atlas Liver Hepatocellular Carcinoma (TCGA‐LIHC) were investigated for the overall/progression‐free survival analysis of GDF2 (BMP9)‐altered HCC patients in comparison with GDF2 (BMP9)‐unaltered HCC patients, and the correlation analysis of the mRNA expression between GDF2 (BMP9) and EPCAM.

### 2.16 Statistical analysis

Student's *t*‐test, one‐way ANOVA, chi‐square test, or Fisher's exact test, and log‐rank test were performed using graphpad prism 7 (GraphPad Software, San Diego, CA, USA). A *P* value of less than 0.05 was considered significant.

## 3 Results

### 3.1 Elevated BMP9 expression is associated with poorer HCC prognosis

**Fig. 1:**
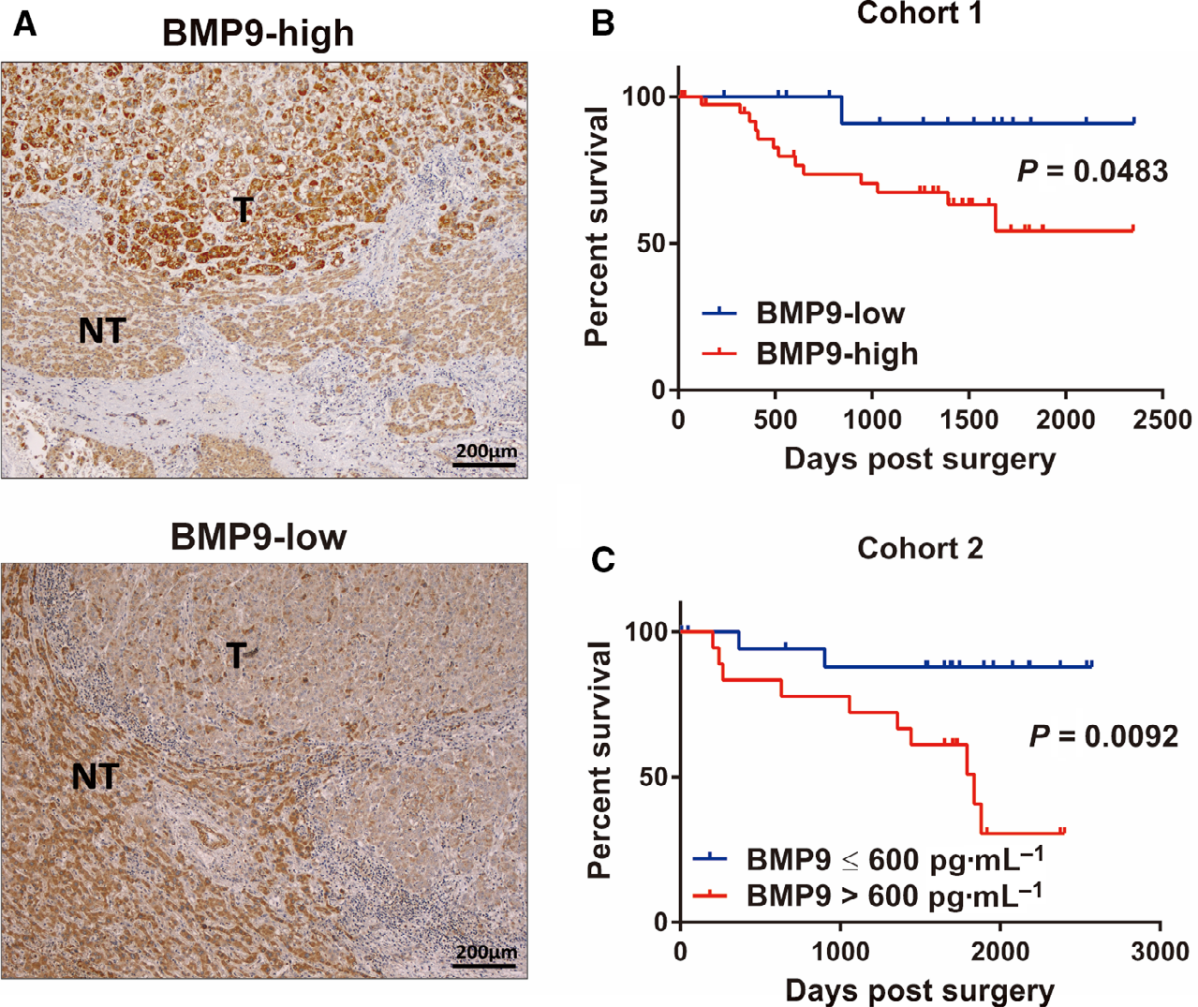
High expression of BMP9 is associated with poor prognosis of HCC patients. (A) Representative IHC staining images of BMP9‐high tumor (upper panel) and BMP9‐low tumor (lower panel) in surgically resected HCC tissue specimens. T, tumor tissue; NT, nontumor tissue. Scale bar = 200 μm. (B) Kaplan–Meier survival curves according to BMP9 expression in HCC tissue specimens (cohort 1, *n* = 54). BMP9‐high (*n* = 38), BMP9‐low (*n* = 16). (C) Kaplan–Meier survival curves according to BMP9 expression in the serum of HCC patients (cohort 2, *n* = 37). BMP9 > 600 pg·mL^−1^ (*n* = 18), BMP9 ≤ 600 pg·mL^−1^ (*n* = 19). Survival curves of two groups were compared using the log‐rank test. [Colour figure can be viewed at wileyonlinelibrary.com]

To examine the relationship between BMP9 expression in HCC tissue specimens and patient prognosis, we analyzed BMP9 expression in HCC tissues of 54 patients (cohort 1). IHC staining revealed 38 HCC specimens with elevated BMP9 expression in HCC tissues compared with adjacent liver tissues, and 16 cases with reduced BMP9 expression in HCC tissues versus adjacent tissues (Fig. 1A). To further identify the contribution of BMP9 expression in the prognosis of patients with HCC, we divided these 54 patients into two groups based on BMP9 expression in tumor compared with expression in adjacent liver tissue and analyzed overall survival in these two groups. Interestingly, patients with BMP9‐high HCC exhibited a poorer overall survival than patients with BMP9‐low HCC (*P* = 0.0483, Fig. 1B). On comparative analysis of clinicopathological characteristics in these two groups, BMP9‐high HCC cases showed more recurrence of multiple tumors in the liver (Table 1). Exogenous BMP9 circulates in serum in an active mature form [14], so we further examined the association between serum BMP9 expression and prognosis. When setting the cutoff value of serum BMP9 to 600 ng·mL^−1^, patients with high serum BMP9 (*n* = 19) showed poorer overall survival than patients with low serum BMP9 (*n* = 19) (*P* = 0.0092, Fig. 1C). Although there was no exact match of BMP9 expression between tumor and serum (Fig. S1), these data indicate that high BMP9 expression in tumor tissue or serum is related to dismal overall survival and could be regarded as a prognostic biomarker of HCC.

**Table 1:** Clinicopathological characteristics of BMP9‐high and BMP9‐low HCC patients (cohort 1). AFP, alpha‐fetoprotein; BCLC, Barcelona Clinic Liver Cancer; B, hepatitis B surface (HBs) antigen‐positive; C, hepatitis C virus (HCV) antibody‐positive; NBNC, HBs antigen‐negative and HCV antibody‐negative; NA, not assessed.

Clinicopathological characteristic	BMP9 high (*n* = 38)	BMP9 low (*n* = 16)	*P* value^a^
Mean age, years (range)	67.0 (37–83)	69.5 (37–87)	0.10
Sex, M/F	30/8	11/5	0.42
Median AFP level, ng·mL^−1^ (range)	14 (1–54260)	10 (3–28340)	0.91
Histological grade^b^: I/II/III/IV	3/15/19/1	1/9/6/0	0.67
Median tumor size, cm (range)	2.9 (1.2–16.5)	3.2 (1.4–14.5)	0.73
Microscopic portal invasion, *N* (%)	19 (50%)	5 (31.3%)	0.21
BCLC stage: 0/A/B/C	4/27/6/1	0/13/2/1	0.51
Virus status: B/C/NBNC	14/12/12	2/6/8	0.18
The stage of liver fibrosis^c^: 0/1/2/3/4/NA	4/1/2/7/22/1	2/1/3/3/5/2	0.36
EpCAM staining: positive/negative	21/17	3/13	0.03
CD90 staining: positive/negative	16/22	6/10	0.75
Recurrence rate, *N* (%)	24 (63.2)	10 (62.5)	0.96
Recurrence pattern: Single/multiple/extrahepaptic	7/16/1	7/3/0	0.03

^a^ Student's *t*‐test, chi‐square test, or Fisher's exact test.

^b^ Edmondson–Steiner grading.

^c^ New Inuyama classification.

### 3.2 BMP9 promotes EpCAM+ HCC‐CSC properties

Our clinical data suggest that BMP9 is potentially involved in the malignant nature of HCC; hence, we confirmed the relevance between BMP9 and HCC‐CSC subtype clinically. By the evaluation of EpCAM/CD90 staining in BMP9‐high/‐low HCC specimens (Fig. S2A,B), we found that EpCAM‐positive ratio is higher in BMP9‐high HCC than that in BMP9‐low HCC, while there is no difference in CD90‐positive ratio between BMP9‐high HCC and BMP9‐low HCC (Table 1). We further confirmed the correlation between BMP9 and EpCAM in a public dataset TCGA‐LIHC. In this dataset, GDF2 (BMP9) alteration was correlated with the overall/progression‐free survival of HCC patients (Fig. S2C,D). By analyzing the correlation of mRNA expression, GDF2 (BMP9) was correlated slightly with EPCAM in the dataset (Fig. S2E). These findings imply that BMP9 is associated with EpCAM+ HCC‐CSCs.

**Fig. 2:**
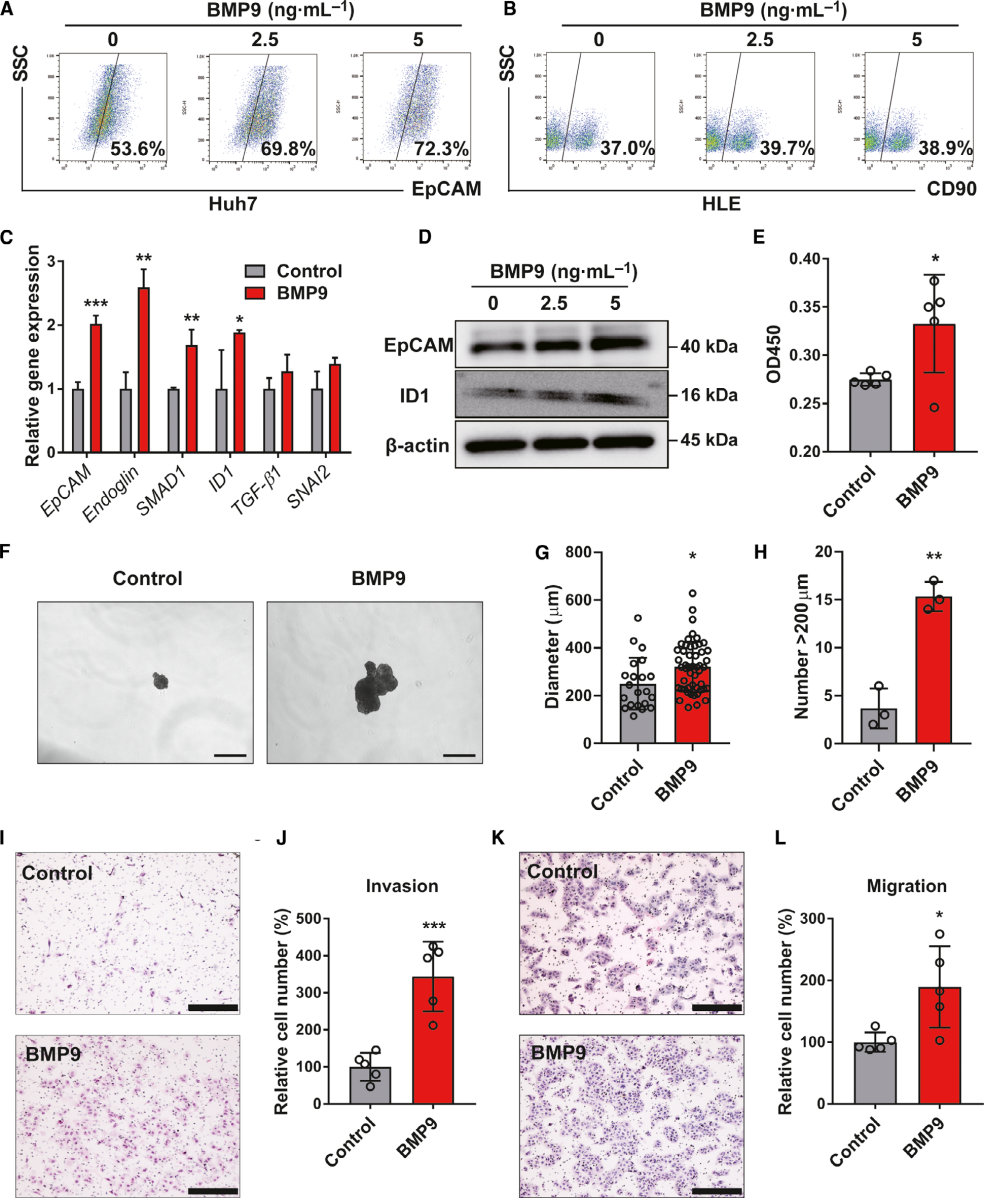
BMP9 promotes EpCAM+ CSC properties in HCC cells. (A, B) Flow cytometry analysis of the percentage of EpCAM‐positive cells in Huh7 cells and the percentage of CD90‐positive cells in HLE cells. Cells were treated with dimethyl sulfoxide or BMP9 (2.5 ng·mL^−1^, 5 ng·mL^−1^) for 10 days. (C) Relative gene expression levels of EpCAM, Endoglin, SMAD1, ID1, TGF‐β1, and SNAI2 in Huh7 cells treated with BMP9 (2.5 ng·mL^−1^) for 5 days. (D) Western blot analysis of EpCAM and ID1 expression in Huh7 cells treated with different concentrations of BMP9 for 5 days. (E) Optical density at 450 nm (OD450) in cell proliferation assay of Huh7 cells treated with BMP9 (5 ng·mL^−1^) for 48 h. (F) Representative spheroid assay images of Huh7 cells treated with or without BMP9 (5 ng·mL^−1^) for 14 days. BMP9 was added to the medium twice a week. Scale bar = 500 μm. (G) Spheroid diameter of (F). (H) Number of spheroids measuring > 200 µm of (F). (I) Representative invasion assay images of Huh7 cells treated with BMP9 (5 ng·mL^−1^) for 24 h. Scale bar = 200 μm. (J) Relative cell numbers from invasion assay. (K) Representative migration assay images of Huh7 cells treated with BMP9 (5 ng·mL^−1^) for 24 h. Scale bar = 200 μm. (L) Relative cell numbers from migration assay. Error bars represent the SD from at least three independent biological replicates. Student's *t*‐test was used to calculate *P* values represented as *P < 0.05; **P < 0.01; ***P < 0.001. [Colour figure can be viewed at wileyonlinelibrary.com]

Given clinical findings, we investigated the functional roles of BMP9 in the regulation of HCC‐CSCs *in vitro*. Flow cytometry analysis showed that BMP9 increases EpCAM+ cells in Huh7 in a dose‐dependent manner, but does not increase CD90+ cells in HLE cells (Fig. 2A,B). BMP9 upregulated the gene expression of EpCAM and BMP9 signaling genes including Endoglin, SMAD1, and ID1 in Huh7 (Fig. 2C). In contrast, BMP9 did not increase the expression of THY1 (CD90) and BMP9 signaling genes in HLE cells (Fig. S3A). In addition, BMP9 does not increase EpCAM and ID1 expression in CD90+ (EpCAM‐negative) cell lines (Fig. S3B). These data suggested that BMP9 promotes the malignant nature of HCC through the regulation of EpCAM+ CSCs, not CD90+ CSCs. Consistent with this, western blot analysis demonstrated that BMP9 increases EpCAM and ID1 expression in EpCAM+ cell lines in a dose‐dependent manner (Fig. 2D and Fig. S3C). In addition, BMP9 promoted cell proliferation, spheroid formation, and the ability of invasion/migration in EpCAM+ HCC cell line Huh7 and MT (Fig. 2E–L and Fig. S3D–G). Taken together, BMP9 promotes HCC‐CSC properties with the activation of EpCAM.

### 3.3 Inhibition of ID1 represses the BMP9‐induced HCC‐CSC properties

**Fig. 3:**
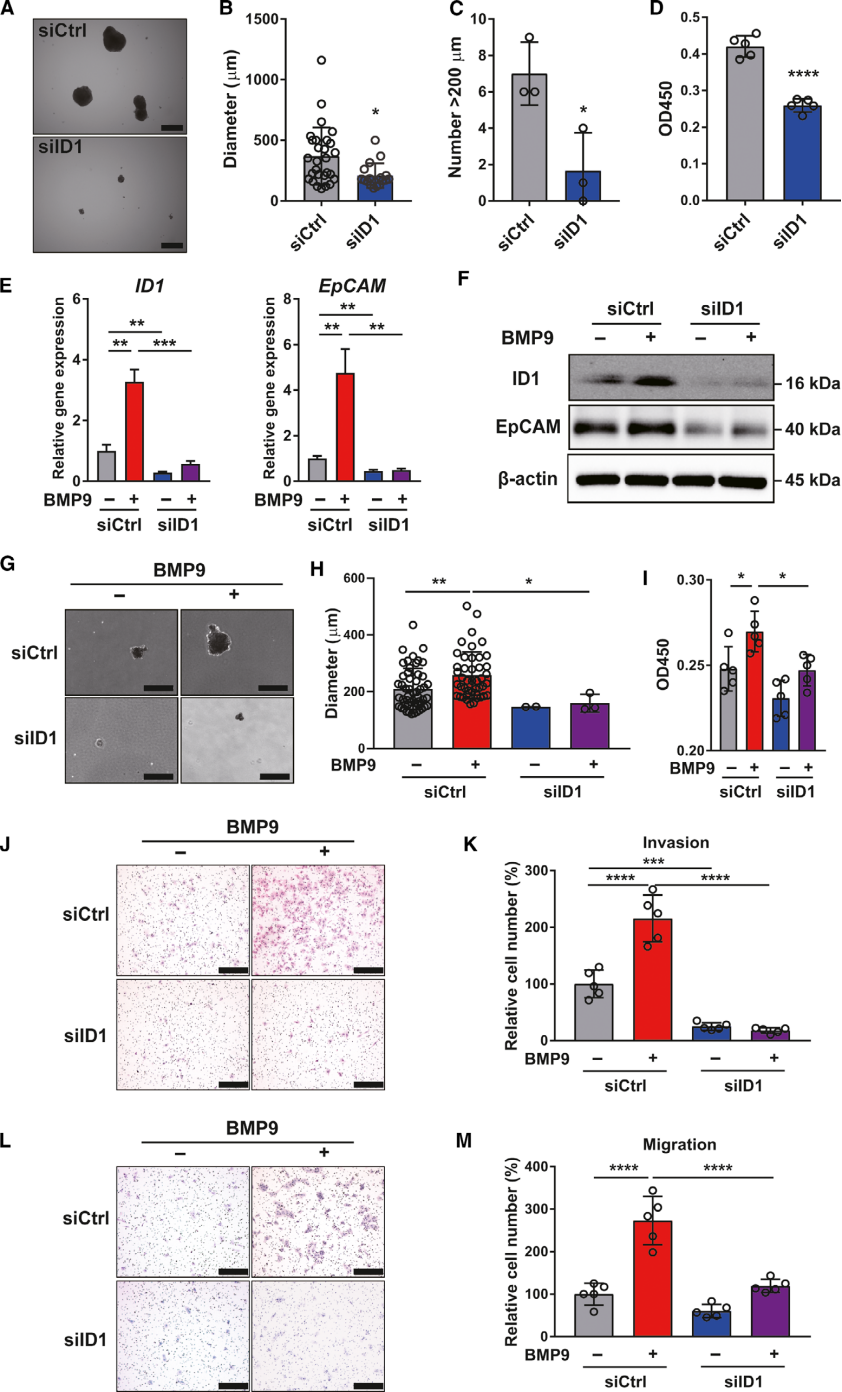
Inhibition of ID1 represses BMP9‐induced HCC‐CSC properties. (A) Representative spheroid assay images of Huh7 cells transfected with control siRNA (siCtrl) or ID1 siRNA (siID1). Scale bar = 500 µm. (B) Spheroid diameter of (A). (C) Number of spheroids measuring > 200 µm of (A). (D) OD450 in cell proliferation assay of siCtrl or siID1 transfected Huh7 cells. (E) Relative gene expression levels of ID1 and EpCAM in Huh7 cells treated with or without BMP9 (5 ng·mL^−1^) for 48 h following siRNA transfection. (F) Western blot analysis of ID1 and EpCAM expression in Huh7 cells treated with or without BMP9 (5 ng·mL^−1^) for 48 h following siRNA transfection. (G) Representative spheroid assay images of Huh7 cells treated with or without BMP9 (5 ng·mL^−1^) for 14 days following siRNA transfection. BMP9 was added to the medium twice a week. Scale bar = 500 μm. (H) Spheroid diameter of (G). (I) OD450 in cell proliferation assay of Huh7 cells treated with or without BMP9 (5 ng·mL^−1^) for 24 h following the siRNA transfection. (J) Representative invasion assay images of Huh7 cells treated with or without BMP9 (5 ng·mL^−1^) for 24 h following siRNA transfection. Scale bar = 200 μm. (K) Relative cell number from invasion assay. (L) Representative migration assay images of Huh7 cells treated with or without BMP9 (5 ng·mL^−1^) for 24 h following the siRNA transfection. Scale bar = 200 μm. (M) Relative cell number from migration assay. Error bars represent the SD from at least three independent biological replicates. Student's *t*‐test was used to calculate *P* values represented as *P < 0.05, **P < 0.01, ***P < 0.001, ****P < 0.0001. [Colour figure can be viewed at wileyonlinelibrary.com]

It has been reported that ID1 is a known BMP9 target gene in HCC cells [10, 15]. In our study, we found that ID1 expression is correlated with BMP9 expression clinically (Fig. S4A,B). In EpCAM+ and CD90+ HCC cell lines, there was no significant difference in the expression of BMP9 among diverse HCC cell lines, while ID1 was highly expressed in EpCAM+ cell lines (Fig. S4C). This implies that BMP9 regulates EpCAM+ HCC‐CSCs via the upregulation of ID1. Since BMP9 promoted not only ID1, but also other ID proteins (including ID2, ID3, and ID4) (Fig. S4D), we investigated whether ID1 and other ID proteins are related to the regulation of EpCAM+ HCC‐CSCs. In the siRNA knockdown assay, we found that among all the ID proteins, only ID1 regulates EpCAM expression in Huh7 cells (Fig. S4E). Based on this, we mainly evaluated the involvement of ID1 enhanced by BMP9 in the regulation of EpCAM+ CSC phenotypes. As expected, ID1 knockdown significantly inhibited spheroid formation (Fig. 3A–C and Fig. S5A–C) and cell proliferation (Fig. 3D). More importantly, ID1 knockdown suppressed BMP9‐induced ID1 and EpCAM expression (Fig. 3E,F) as well as BMP9‐induced spheroid formation (Fig. 3G,H), cell proliferation (Fig. 3I), and invasion/migration ability (Fig. 3J–M) in Huh7 cells. We additionally confirmed that silencing of ID1 reduces BMP9‐induced EpCAM+ HCC‐CSC properties in MT cells (Fig. S5D–K). These findings suggest that ID1, which is activated by BMP9, is the pivotal transcription factor regulating EpCAM+ HCC‐CSCs.

### 3.4 BMP9‐ID1 cooperatively regulates EpCAM+ HCC‐CSCs through activating Wnt/β‐catenin signaling

**Fig. 4:**
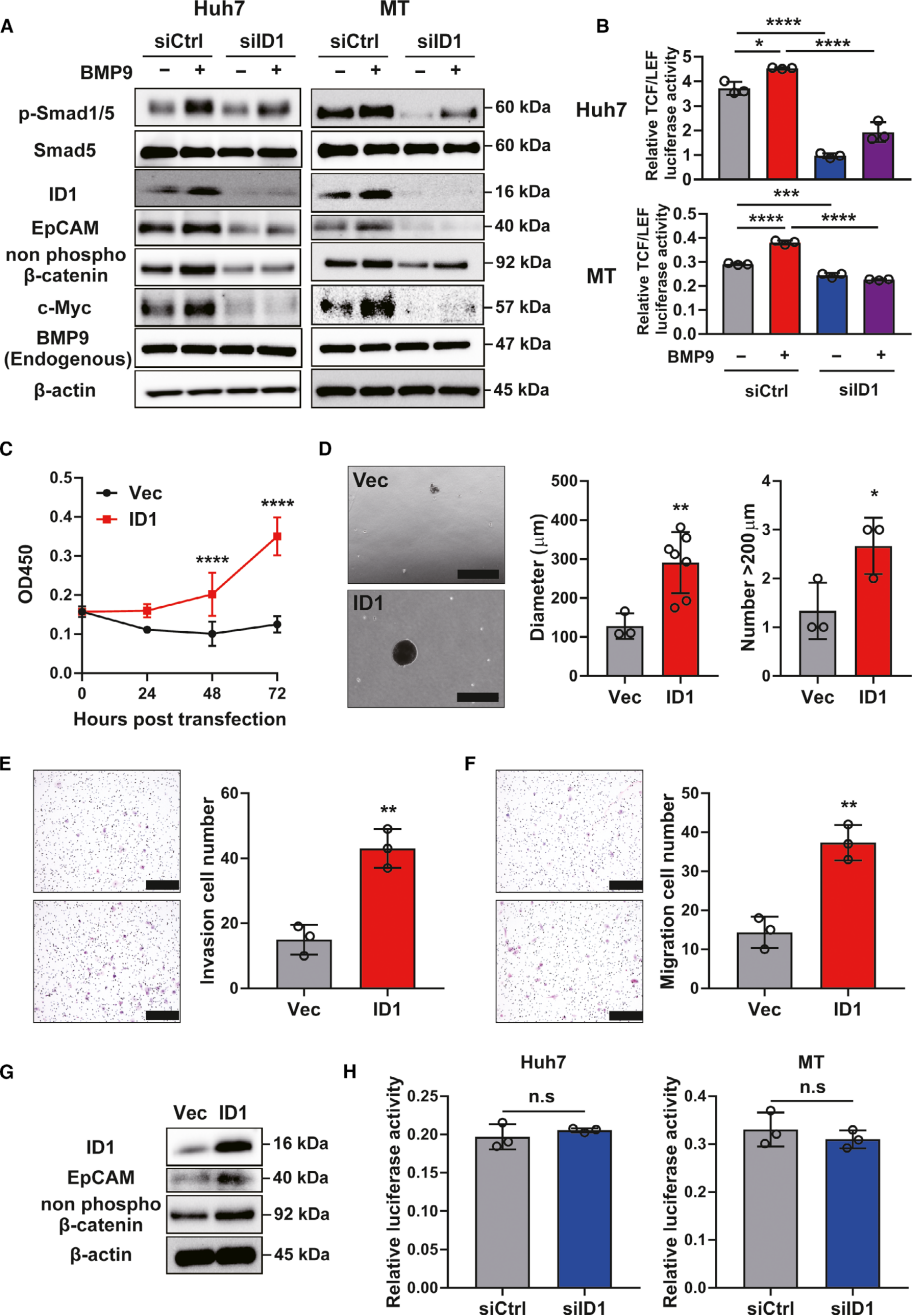
BMP9‐ID1 cooperatively regulates EpCAM+ HCC‐CSCs through activating Wnt/β‐catenin signaling. (A) Western blot images of ID1 knockdown Huh7/MT cells treated with or without BMP9. (B) Relative TCF/LEF luciferase activity in ID1 knockdown Huh7/MT cells treated with or without BMP9. (C) OD450 in cell proliferation assay of control vector (Vec) or ID1 vector (ID1)‐transfected MT cells. (D) Representative spheroid assay images, spheroid diameter, and number of spheroids (> 200 µm) of MT cells transfected with Vec or ID1. Scale bar = 500 µm. (E) Representative invasion assay images and relative cell numbers from invasion assay of MT cells transfected with Vec or ID1. Scale bar = 200 µm. (F) Representative migration assay images and relative cell numbers from migration assay of MT cells transfected with Vec or ID1. Scale bar = 200 µm. (G) Western blot images of Vec‐ or ID1‐transfected MT cells. (H) Relative luciferase activity in ID1 knockdown Huh7 and MT cells transfected with pGL4.10 containing 2151bp promoter/enhancer fragment of the EpCAM gene. Error bars represent the SD from at least three independent biological replicates. Student's *t*‐test was used to calculate *P* values represented as *P < 0.05, **P < 0.01, ***P < 0.001, ****P < 0.0001. n.s, not significant. [Colour figure can be viewed at wileyonlinelibrary.com]

In terms of understanding the mechanism of how BMP9‐ID1 regulates EpCAM+ HCC‐CSCs, we investigated the interaction between BMP9‐ID1 and Wnt/β‐catenin signaling, which is reported as the regulator of EpCAM+ HCC‐CSCs [16]. We found that BMP9 not only induces BMP9 signaling molecules (phospho‐SMAD1/5 and ID1) and EpCAM, but also induces non‐phospho‐β‐catenin and c‐Myc, which is a target molecule of Wnt/β‐catenin signaling. Furthermore, silencing of ID1 inhibited phospho‐Smad1/5 and Wnt/β‐catenin signaling, which are induced by BMP9 (Fig. 4A). By assessing Wnt/β‐catenin activity using the TCF/LEF luciferase reporter system, we further confirmed that silencing of ID1 can suppress BMP9‐induced the Wnt/β‐catenin activity (Fig. 4B). In contrast, overexpression of ID1 greatly induced the cell proliferation, spheroid formation, and invasion/migration ability with the expression of non‐phospho‐β‐catenin and c‐Myc as well as EpCAM (Fig. 4C–G).

By investigating the direct role of ID1 in the activation of the EpCAM gene by a luciferase reporter vector containing 2151 bp promoter/enhancer fragment of the EpCAM gene, we found that there is no significant difference in the promoter/enhancer activity of EpCAM gene between ID1 siRNA‐transfected cells and control siRNA‐transfected cells (Fig. 4H). We further confirmed that there is no putative binding site of ID1 to the EpCAM promoter by using ALGGEN PROMO, a database to predict transcription factor binding sites in DNA sequences (data not shown). These findings imply that ID1 does not directly activate the EpCAM expression via binding the promoter of EpCAM gene. Taken together, our data comprehensively suggest that BMP9‐ID1 cooperatively regulates EpCAM+ HCC‐CSCs through activating Wnt/β‐catenin signaling.

### 3.5 BMP receptor inhibitors suppress BMP9‐ID1‐induced HCC‐CSC properties

**Fig. 5:**
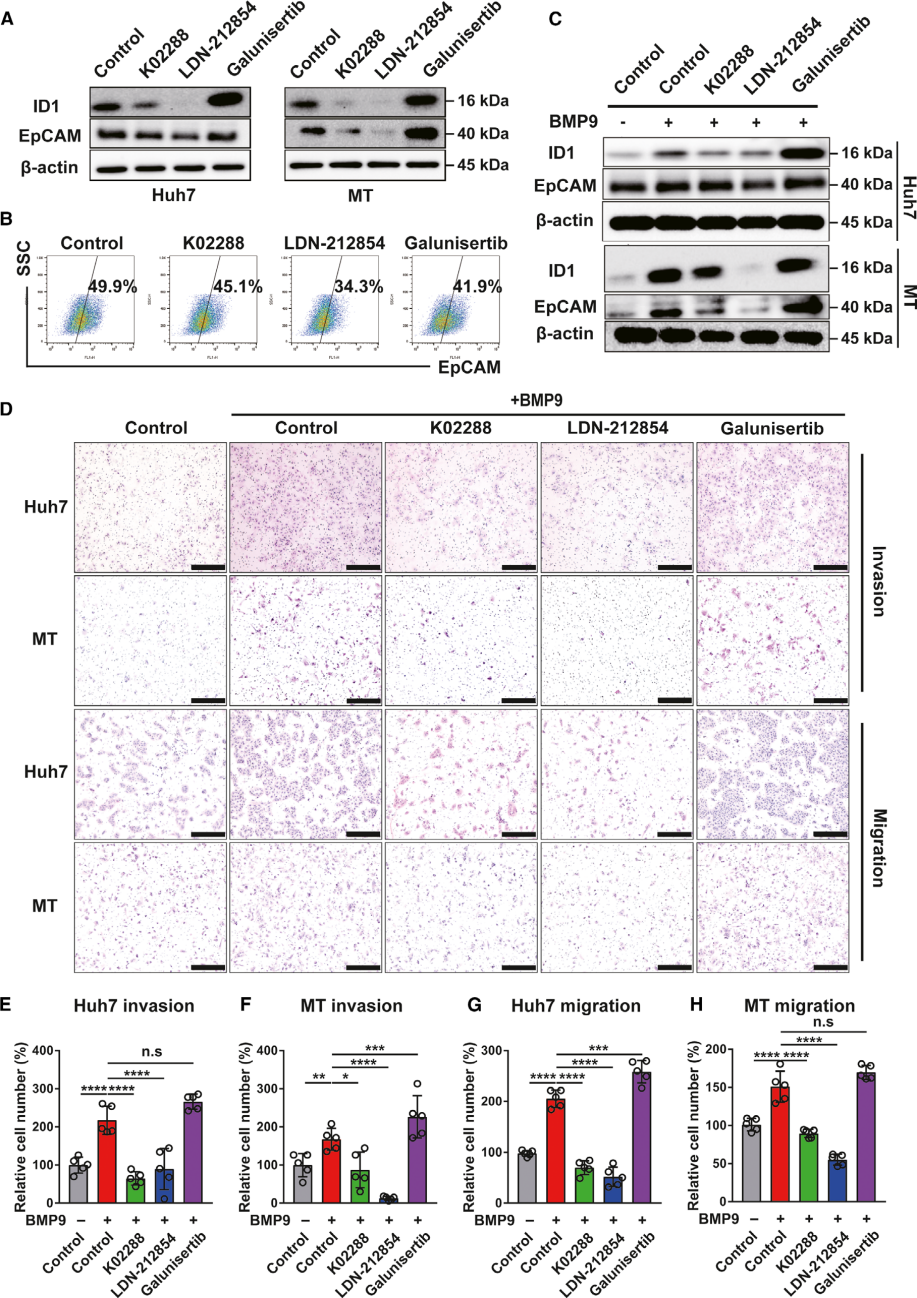
BMP receptor inhibitors suppress BMP9‐ID1 signaling‐induced HCC‐CSC phenotypes. (A) Western blot analysis of ID1 and EpCAM expression in Huh7 and MT cells treated with BMP/TGF‐β receptor inhibitors. (B) Flow cytometry analysis of EpCAM‐positive Huh7 cells treated with BMP/TGFβ receptor inhibitors (0.25 μm) for 5 days. The percentage indicates EpCAM‐positive cells. (C) Western blot analysis of ID1 and EpCAM expression in Huh7 cells treated with BMP/TGF‐β receptor inhibitors (0.5 μm) in the presence of BMP9 (5 ng·mL^−1^) for 48 h. (D) Representative invasion/migration assay images of Huh7 and MT cells treated with BMP/TGF‐β receptor inhibitors (1 μm) in the presence of BMP9 (10 ng·mL^−1^) for 24 h. Scale bar = 200 μm. (E–H) Relative cell number of invasion/migration assay in Huh7 and MT cells. Error bars represent the SD from five independent biological replicates. One‐way ANOVA was used to calculate *P* values represented as *P < 0.05, **P < 0.01, ***P < 0.001, ****P < 0.0001. n.s, not significant. [Colour figure can be viewed at wileyonlinelibrary.com]

The above data indicated that BMP9‐ID1 signaling could be a crucial therapeutic target for suppressing HCC‐CSCs. Thus, we examined the effect of the BMP receptor inhibitors K02288 and LDN‐212854. Compared with the TGF‐β receptor inhibitor galunisertib, the IC50 value of the BMP receptor inhibitors tended to be lower in Huh7 and MT cells (Table S2 and Fig. S6A,B). In addition, BMP9 receptor inhibitors, especially LDN‐212854, suppressed not only ID1 and EpCAM expression but also cell proliferation, while galunisertib suppressed neither (Fig. 5A,B and Fig. S6C,D). These findings implied that endogenous BMP9 upregulates ID1 and EpCAM through BMP receptor signaling and not TGF‐β receptor signaling. We then evaluated the effect of BMP/TGF‐β receptor inhibitors in the presence of BMP9. As expected, K02288 and LDN‐212854 significantly suppressed the BMP9‐induced upregulation of ID1 and EpCAM expression (Fig. 5C), as well as cell invasion/migration ability (Fig. 5D–H). Taken together, these findings suggest that BMP receptor inhibitors are potentially promising therapeutic agents against HCC‐CSCs.

### 3.6 BMP9 receptor inhibitors suppress HCC tumor progression through repression of ID1 *in vivo*

**Fig. 6:**
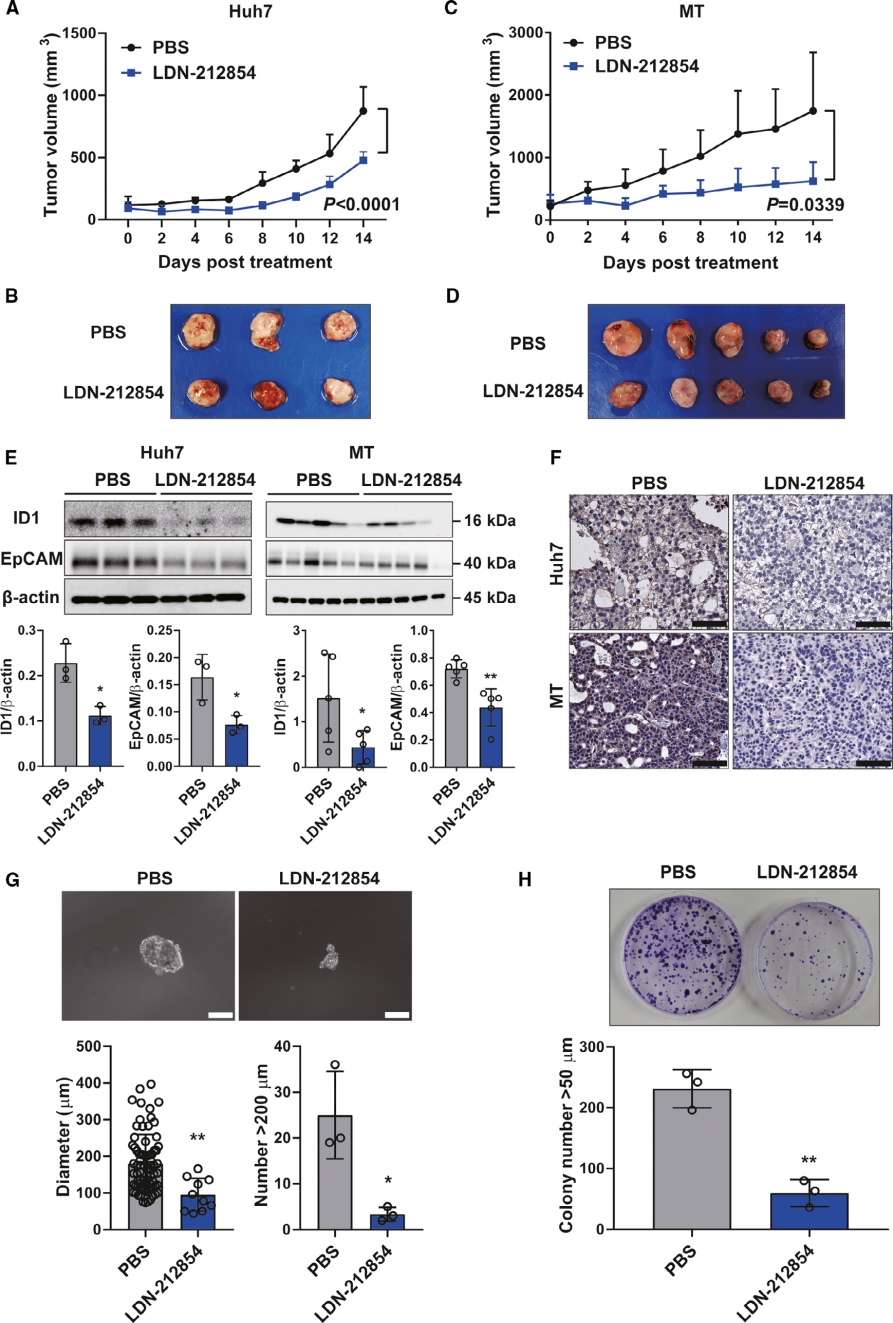
BMP receptor inhibitor LDN‐212854 suppresses tumor growth of HCC xenografts through repression of ID1 *in vivo*. (A) Effect of LDN‐212854 on growth of Huh7 cells. (B) Xenograft tumors of Huh7 cells treated with PBS (*n* = 3) or LDN‐212854 (*n* = 3). (C) Effect of LDN‐212854 on growth of MT cells. (D) Xenograft tumors of MT cells treated with PBS (*n* = 5) or LDN‐212854 (*n* = 5). (E) Western blot analysis of ID1 and EpCAM expression in xenograft tumor of Huh7 and MT cells. β‐actin was used as the reference for quantifying the protein expression. (F) Representative IHC staining images of ID1 expression in xenografts of Huh7 and MT cells. Scale bar = 200 µm. (G) Representative spheroid assay images of Huh7 xenografts treated with PBS or LDN‐212854. Scale bar = 200 µm. Spheroid diameter (µm) and number of spheroids measuring > 200 µm in each group were shown. (H) Representative colony formation assay images of Huh7 xenografts treated with PBS or LDN‐212854. The colony numbers measuring > 50 µm in each group were shown. Error bars represent the SD from at least three independent biological replicates. Student's *t*‐test was used to calculate *P* values represented as *P < 0.05, **P < 0.01. [Colour figure can be viewed at wileyonlinelibrary.com]

Of the BMP/TGF‐β receptor inhibitors evaluated *in vitro*, LDN‐212854 was the most potent to suppress BMP9‐ID1 signaling and CSC properties, and thus, we selected LDN‐212854 as the inhibitor for assessing the antitumor effect on HCC xenografts. Compared with PBS, LDN‐212854 significantly suppressed tumor growth in Huh7 cells (*P* < 0.0001) and MT cells (*P* = 0.0339) *in vivo* mouse xenograft model (Fig. 6A–D) without severe toxicity (Fig. S7A). In addition, LDN‐212854 inhibited ID1 and EpCAM expression in tumor tissue specimens (Fig. 6E,F and Fig. S7B). In addition, *ex vivo* limiting dilution assay and colony formation assay showed that LDN‐212854‐treated tumor cells have less spheroid/colony formation ability than PBS‐treated tumor cells (Fig. 6G,H). These data clearly demonstrated the effect of the BMP receptor inhibitor LDN‐212854 on the inhibition of HCC tumor growth via suppressing BMP9‐ID1 signaling, which promotes EpCAM+ CSC properties, suggesting that targeting BMP9‐ID1 signaling could be a promising therapeutic option for HCC patients.

## 4 Discussion

The malignant nature of HCC is now thought to be associated with the presence of CSCs, and therefore, the development of therapeutic options against CSCs is strongly desired. Bone morphogenetic protein 9 (BMP9), a member of the BMP subgroup of the TGF‐β superfamily proteins that signal via type I and type II BMP receptors, is involved in osteogenesis, angiogenesis, and liver development [8, 17]. BMP9 has recently been reported to be associated with liver diseases including cancer [15, 18]. However, unlike TGF‐β, which has been reported to be associated with HCC‐CSCs [19, 20, 21], the relationship between BMPs and HCC‐CSCs has been unclear. We therefore attempted to clarify the role of BMP9 signaling pathway in the regulation of the malignant nature of HCC.

In this study, we first found that high expression of BMP9 in tumor tissue or serum is associated with poor prognosis in HCC patients. This suggests that BMP9 is associated with the malignancy of HCC. By the evaluation of EpCAM/CD90 status in BMP9‐high/BMP9‐low HCC specimens and the correlation analysis between BMP9 and EpCAM in a public dataset, we found that BMP9 is associated with EpCAM+ HCC‐CSCs.

*In vitro*, BMP9 increased HCC‐CSCs in EpCAM+ cells with the expression of EpCAM and BMP9 signaling genes, while BMP9 did not increase HCC‐CSCs in CD90+ cells. BMP9 also promoted cell proliferation and invasion/migration ability in EpCAM+ HCC cells. These findings suggest that BMP9 promotes CSC properties in the EpCAM+ HCC subtype. Consistent with this, two independent studies have demonstrated the cell proliferative effect of BMP9 in HepG2, which is also an EpCAM+ HCC cell line [9, 10]. In subsequent examination of the relationship between BMP9 signaling and HCC subtype, we found that the transcription factor ID1, which is a known target of BMP9 signaling, is abundantly expressed in EpCAM+ HCC cell lines. These findings imply that BMP9 is associated with the malignant phenotype of EpCAM+ HCC cells, which overexpress ID1, and BMP9‐ID1 cooperatively regulates CSC properties in HCC. In liver cells, it has been reported that BMP9 transduces its signals through the activation of BMP receptors and the phosphorylation of Smad1/5/8. These Smad proteins play an important role in transducing the signal from BMP receptors to target genes, including ID1, within the cell nucleus. It is interested that ID1 regulates Wnt and SHH signaling in glioblastoma stem cells by suppressing CULLIN3 ubiquitin ligase [22]. In terms of the maintenance of HCC‐CSCs, EpCAM expression is regulated by Wnt/β‐catenin signaling [16]. Based on these reports, we hypothesized that BMP9‐ID1 signaling regulates EpCAM+ HCC‐CSCs through the activation of Wnt/β‐catenin signaling. As expected, ID1 knockdown significantly suppressed EpCAM+ CSC phenotypes and the activity of Wnt/β‐catenin signaling even in the presence of BMP9. In addition, ID1 did not affect the promoter/enhancer of EpCAM gene directly. These findings suggested that BMP9‐ID1 signaling regulates EpCAM+ HCC‐CSCs through the activation of Wnt/β‐catenin signaling. In the present study, intriguingly, ID1 knockdown suppressed the expression of phospho‐Smad1/5. In liver cells, it has been reported that BMP9 transduces its signals through the activation of BMP receptors and the phosphorylation of Smad1/5/8. These Smad proteins play a role in transducing the signal from BMP receptors to target genes, including ID1, within the cell nucleus [15]. Our finding implies an additional mechanistic consideration that ID1 could also regulate upstream BMP9 signaling.

To further understand the role of BMP9‐ID1 signaling in the regulation of HCC‐CSCs, we assessed the effect of BMP receptor inhibitors in comparison with a potent TGFβ receptor I inhibitor galunisertib, which is currently being used in a clinical trial for advanced HCC [23]. Interestingly, compared with galunisertib, BMP receptor inhibitors more effectively inhibited HCC cell proliferation and suppressed ID1. This implies that BMP receptors, not TGF‐β receptors, are essential for endogenous BMP9 signaling to upregulate ID1 and EpCAM. Furthermore, BMP receptor inhibitors successfully suppressed BMP9‐induced ID1 expression and CSC phenotypes in HCC *in vitro* and tumor growth of HCC xenografts *in vivo*. These results suggest that BMP receptor inhibitors suppress tumor growth via inhibiting BMP9‐ID1 signaling, which regulates HCC‐CSCs. Although several therapeutic options have been developed in recent years [24, 25], further improvement is needed in the prognosis of advanced HCC patients. From our findings, BMP receptor inhibitors could be a novel therapeutic option for targeting HCC‐CSCs and could be promising in combination with other cytotoxic agents, molecular targeted therapeutic agents, or immune checkpoint inhibitors for future clinical application. In fact, the therapeutic effect of BMP receptor inhibitor *in vivo* study still needs to be improved. Thus, future development of combination therapy using BMP receptor inhibitors is awaited in the treatment of advanced HCC patients.

In our study, the status of ID1 expression in HCC specimens was not completely consistent with the status of BMP9 expression, and ID1 regulated EpCAM expression even in the absence of BMP9. These findings imply that other factors may be involved in the activation of ID1 in HCC cells. Other BMPs, especially BMP2/4/7, are also reported as molecules to regulate ID1 expression [26, 27]. In addition, c‐Myc also regulates ID1 by a positive feedback loop regulatory mechanism [28]. Considering that some factors activate ID1 independently of BMP9, directly targeting ID1 could be another crucial therapeutic option to overcome malignant HCC. In some of the past studies, the researchers have attempted to inhibit ID1 directly by using peptide‐conjugated antisense oligonucleotides [29] and a peptide aptamer [30]. However, there is no selective ID1 inhibitor at present. Therefore, exploring other ID1 regulators and developing the selective ID1 inhibitors are still needed in the future.

## 5 Conclusions

Our data collectively indicate that BMP9‐ID1 signaling regulates the expression of EpCAM through the activation of Wnt/β‐catenin signaling, resulting in the promotion of EpCAM+ HCC‐CSC properties. Therefore, targeting BMP9‐ID1 signaling could be a novel therapeutic option to overcome the malignant HCC.

## Conflict of interest

The authors declare no conflict of interest.

## Author contributions

HC and KN conceived the project. HC and KN designed and performed experiments, analyzed the data, and wrote the paper. TT analyzed the data. TY, AS, HN, and NI helped to revise the paper. KN, TY, and SK supervised the project. All authors read and approved the final manuscript.

### Peer Review

The peer review history for this article is available at https://publons.com/publon/10.1002/1878‐0261.12963.

## Supporting information


**Fig. S1.** Comparison of serum BMP9 level in BMP9‐high/‐low HCC patients.

**Fig. S2.** Correlation between BMP9 and HCC‐CSC marker.

**Fig. S3.** BMP9 promotes the expression of ID1/EpCAM and CSC properties in EpCAM+ HCC cells.

**Fig. S4.** ID1 is associated with BMP9 expression and regulates EpCAM expression in HCC cells.

**Fig. S5.** Inhibition of ID1 represses the BMP9‐induced CSC properties in MT cells.

**Fig. S6.** BMP receptor inhibitors suppress ID1 expression and cell proliferation more than TGF‐β receptor inhibitor.

**Fig. S7.** Body weight change and EpCAM expression in tumor of PBS or LDN‐212854 treated Huh7 xenograft mice.

**Table S1.** Selectivity of BMP/TGFβ receptor inhibitor.

**Table S2.** IC50 of BMP/TGFβ receptor inhibitor in Huh7 and MT.

## Data Availability

TCG‐LIHC dataset is a publicly available. Survival analysis and correlation analysis data were obtained from cBioPortal (https://www.cbioportal.org/). The data that support the findings of this study are available in list relevant figures/tables and/or the supplementary material of this article.
