# Supplementary figures and images for: BMP9‐ID1 signaling promotes EpCAM‐positive cancer stem cell properties in hepatocellular carcinoma
Source: Mol Oncol. 2021 May 2;15(8):2203–18. doi: 10.1002/1878-0261.12963 (PMC8333780; doi:10.1002/1878-0261.12963)

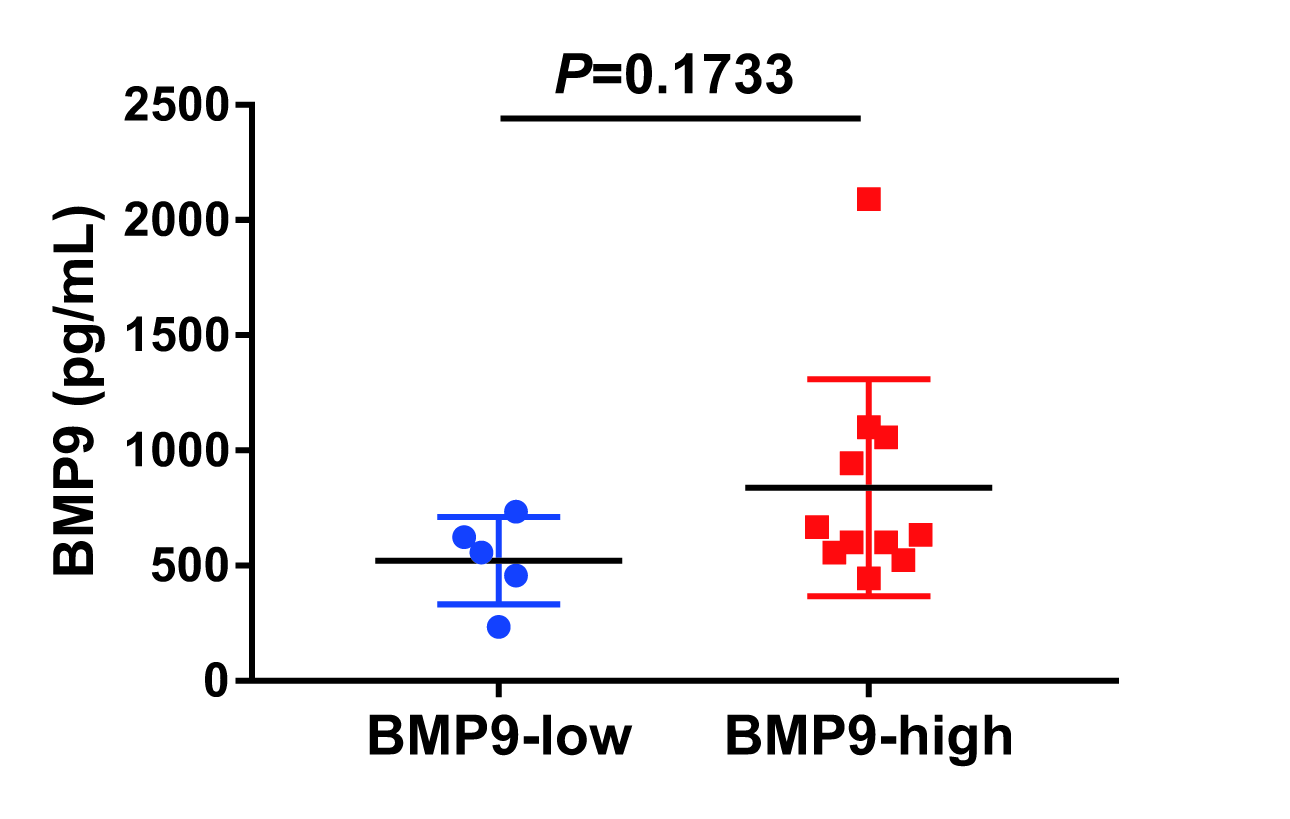

Supplement: Supplementary file 1 — Fig. S1. Comparison of serum BMP9 level in BMP9‐high/‐low HCC patients. [file MOL2-15-2203-s009.tif]

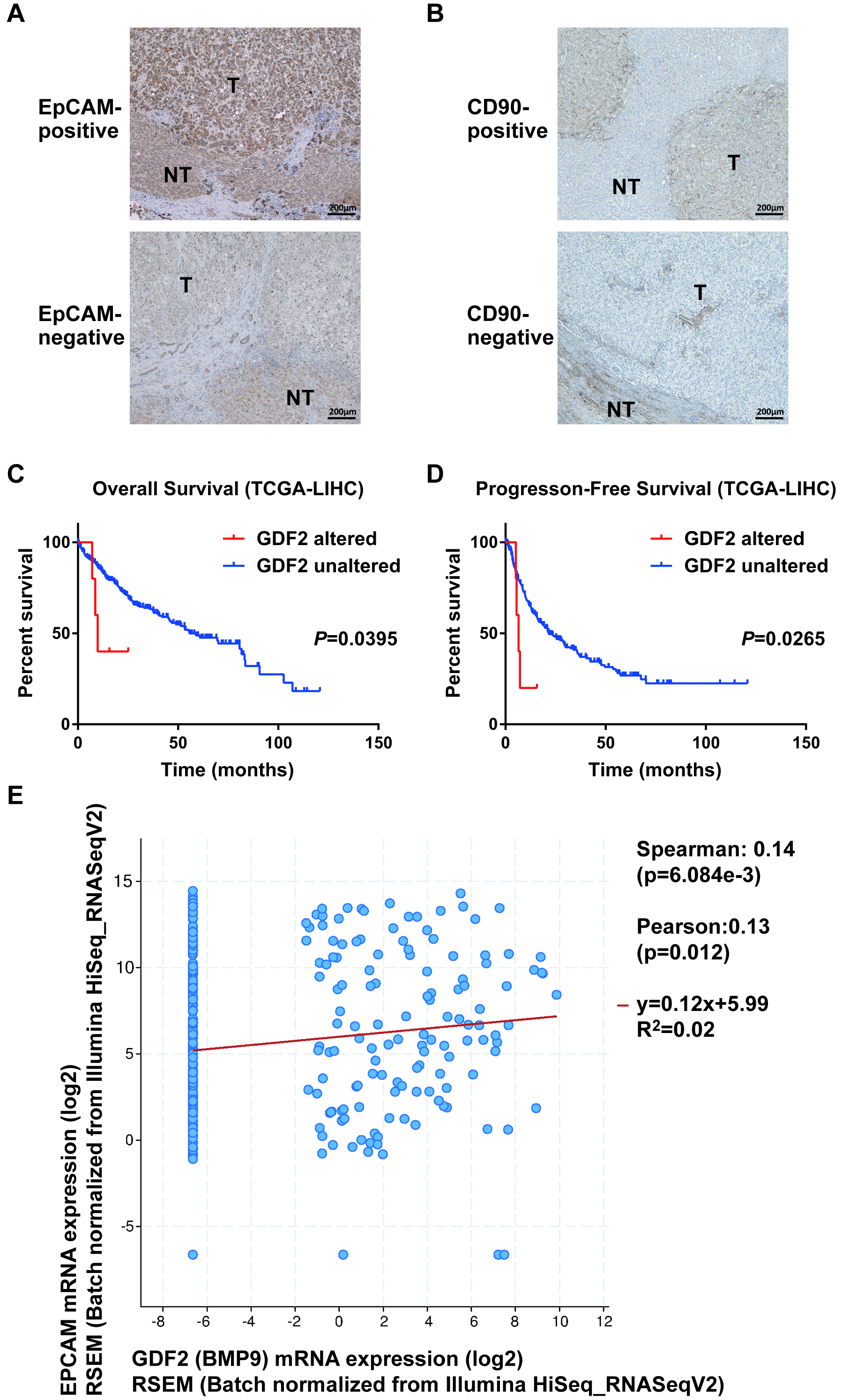

Supplement: Supplementary file 2 — Fig. S2. Correlation between BMP9 and HCC‐CSC marker. [file MOL2-15-2203-s007.tif]

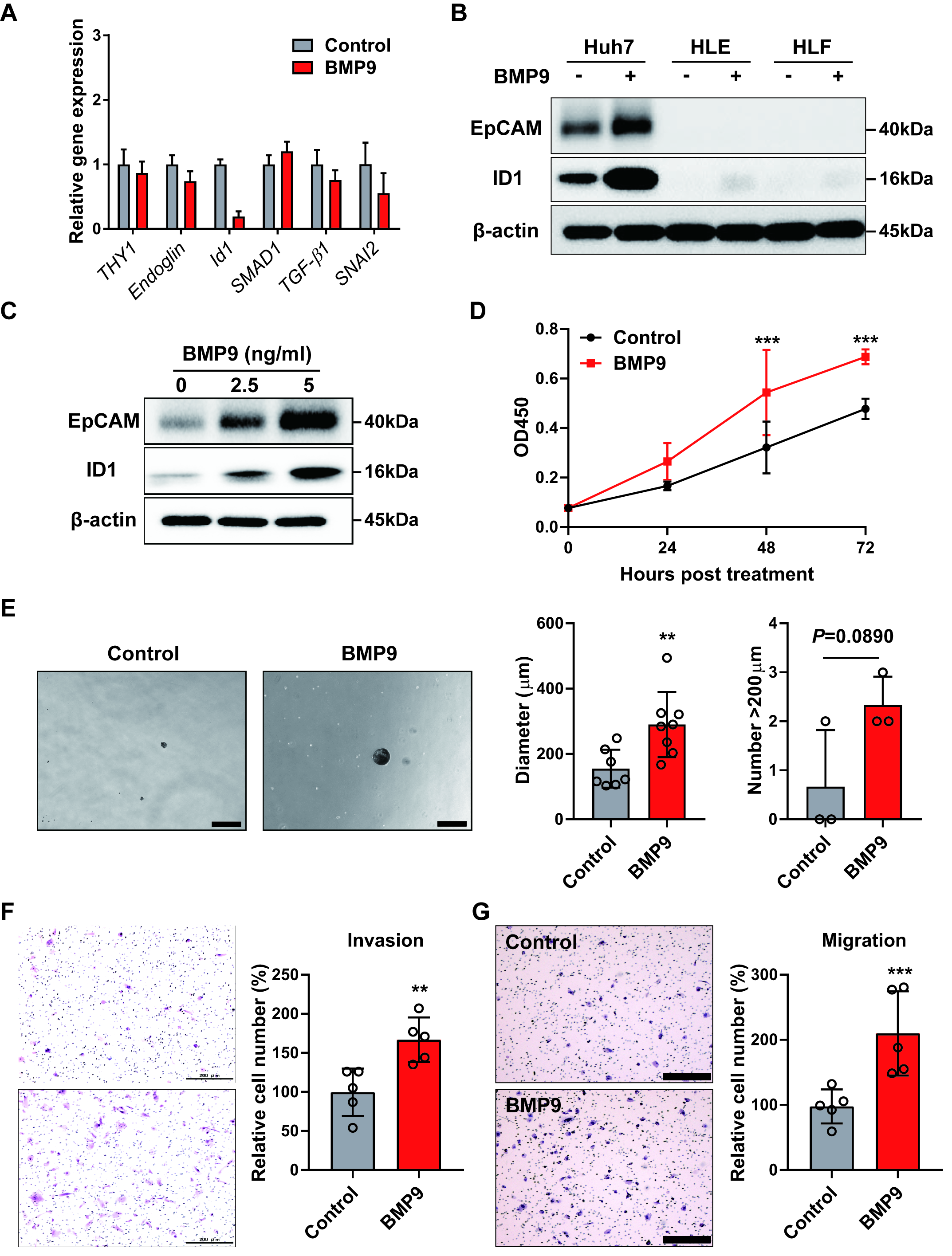

Supplement: Supplementary file 3 — Fig. S3. BMP9 promotes the expression of ID1/EpCAM and CSC properties in EpCAM+ HCC cells. [file MOL2-15-2203-s006.tif]

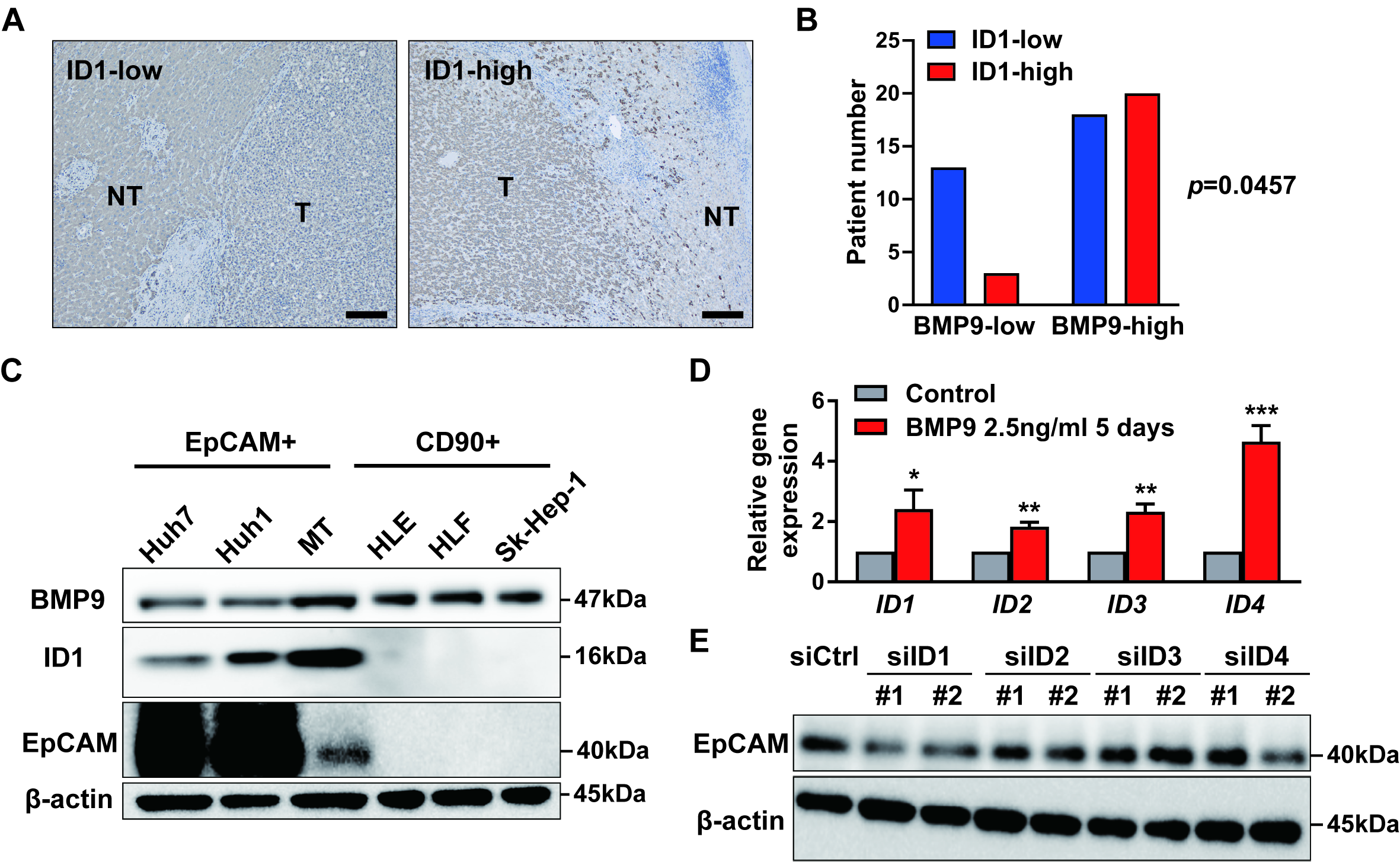

Supplement: Supplementary file 4 — Fig. S4. ID1 is associated with BMP9 expression and regulates EpCAM expression in HCC cells. [file MOL2-15-2203-s001.tif]

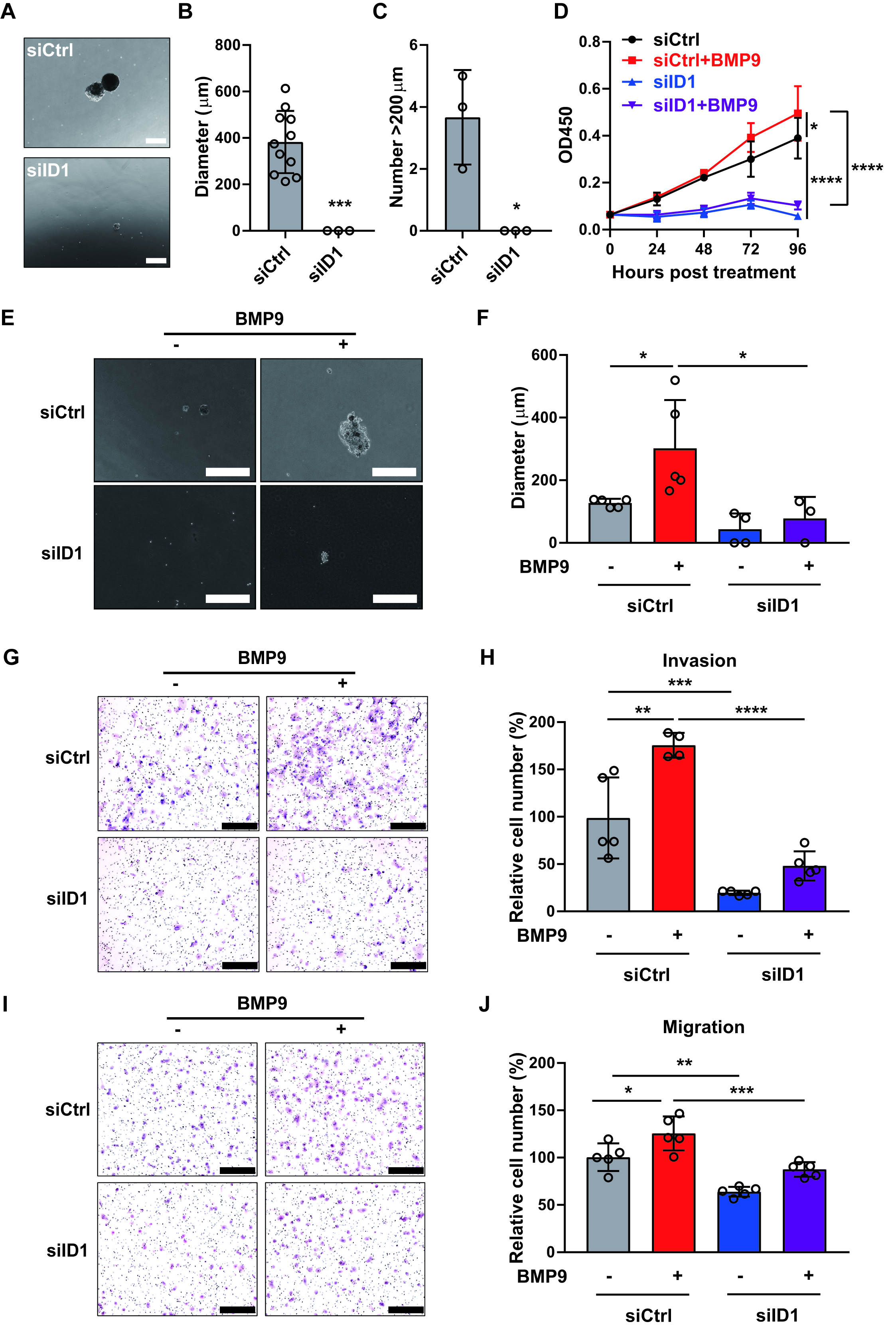

Supplement: Supplementary file 5 — Fig. S5. Inhibition of ID1 represses the BMP9‐induced CSC properties in MT cells. [file MOL2-15-2203-s002.tif]

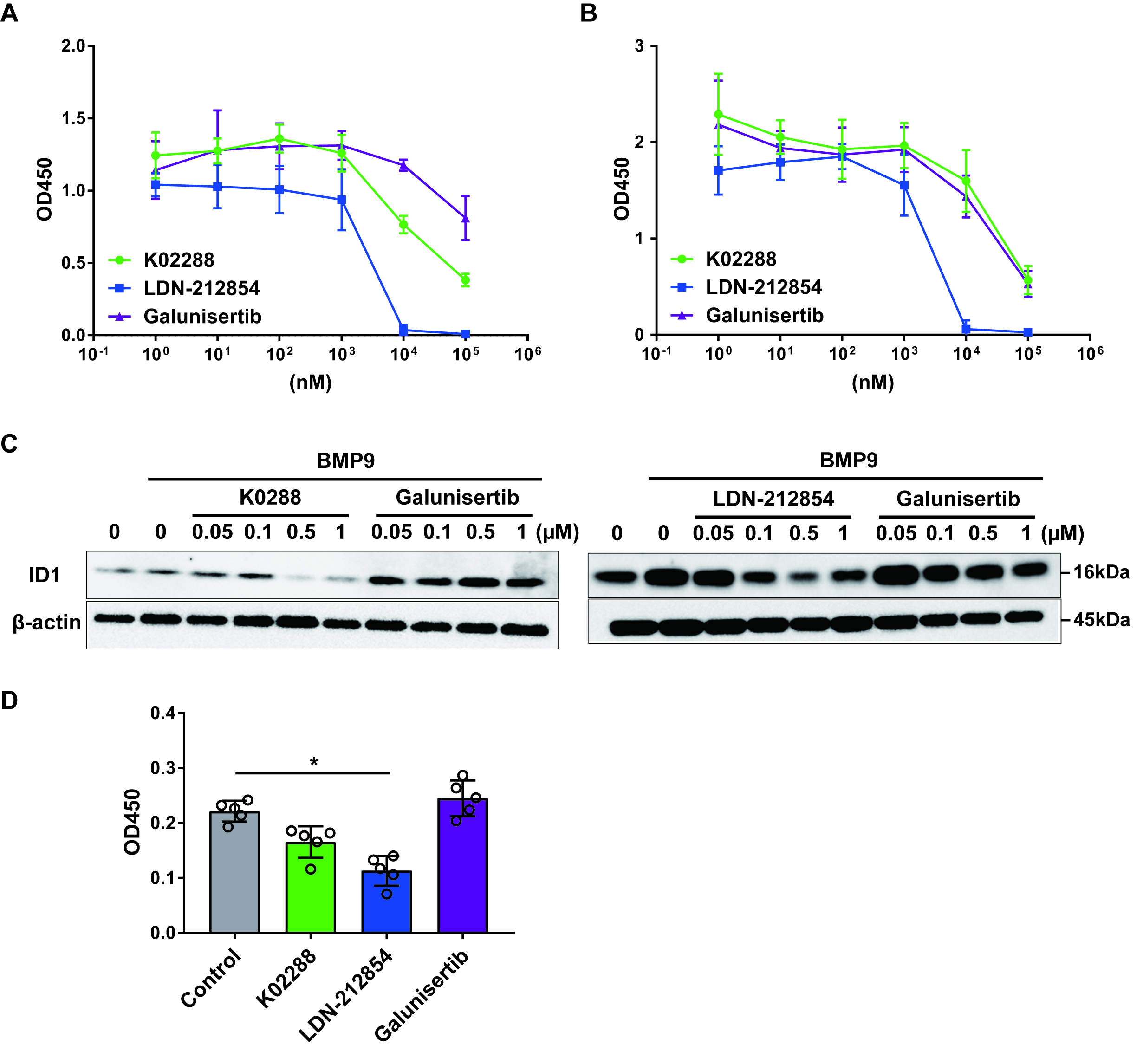

Supplement: Supplementary file 6 — Fig. S6. BMP receptor inhibitors suppress ID1 expression and cell proliferation more than TGF‐β receptor inhibitor. [file MOL2-15-2203-s005.tif]

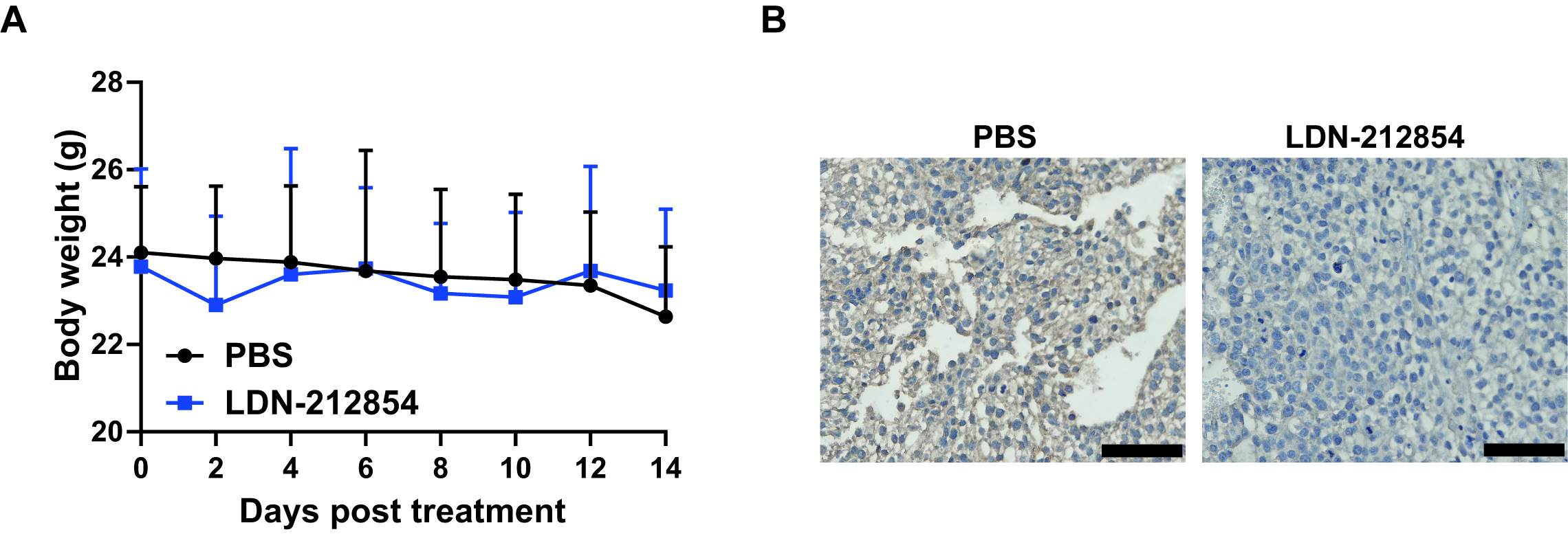

Supplement: Supplementary file 7 — Fig. S7. Body weight change and EpCAM expression in tumor of PBS or LDN‐212854 treated Huh7 xenograft mice. [file MOL2-15-2203-s003.tif]
