# Supplementary material for: BMP9‐ID1 signaling promotes EpCAM‐positive cancer stem cell properties in hepatocellular carcinoma
Source: Mol Oncol. 2021 May 2;15(8):2203–18. doi: 10.1002/1878-0261.12963 (PMC8333780; doi:10.1002/1878-0261.12963)
Supplement: Supplementary file 8 — Table S1. Selectivity of BMP/TGFβ receptor inhibitor. [file MOL2-15-2203-s008.docx]

Supplementary table 1. The selectivity of BMP/TGFβ receptor inhibitor

| Cell-free assay (nM) | BMP/TGFβ receptor | | | | | |
| --- | --- | --- | --- | --- | --- | --- |
|  | ALK1 | ALK2 | ALK3 | ALK4 | TGFβRI/ALK5 | ALK6 |
| K02288 | 1.8 | 1.1 | 34.4 |  |  | 6.4 |
| LDN-212854 | 2.4 | 1.3 | 85.8 | 2133 | 9276 |  |
| Galunisertib |  |  |  |  | 56 |  |
