# Supplementary material for: BMP9‐ID1 signaling promotes EpCAM‐positive cancer stem cell properties in hepatocellular carcinoma
Source: Mol Oncol. 2021 May 2;15(8):2203–18. doi: 10.1002/1878-0261.12963 (PMC8333780; doi:10.1002/1878-0261.12963)
Supplement: Supplementary file 9 — Table S2. IC50 of BMP/TGFβ receptor inhibitor in Huh7 and MT. [file MOL2-15-2203-s004.docx]

Supplementary table 2. IC50 of BMP/TGFβ receptor inhibitor in Huh7 and MT

| Cell line | K02288 (nM) | LDN-212854 (nM) | Galunisertib (nM) |
| --- | --- | --- | --- |
| Huh7 | 9420 | 3468 | 124096 |
| MT | 27282 | 3316 | 20026 |
